# Preparation of Uniform Nano Liposomes Using Focused Ultrasonic Technology

**DOI:** 10.3390/nano13192618

**Published:** 2023-09-22

**Authors:** Ji-Soo Yun, Seon-Ae Hwangbo, Young-Gyu Jeong

**Affiliations:** 1 Nanosafety Team, Safety Measurement Institute, Korea Research Institute of Standards and Science (KRISS), 267 Gajeong-ro, Yuseong-gu, Daejeon 34113, Republic of Korea; jisoo991208@kriss.re.kr; 2 Department of Applied Organic Materials Engineering, Chungnam National University, Daejeon 34134, Republic of Korea

**Keywords:** nano liposome, focused ultrasonication, uniform size distribution, stability

## Abstract

Liposomes are microspheres produced by placing phospholipids in aqueous solutions. Liposomes have the advantage of being able to encapsulate both hydrophilic and hydrophobic functional substances and are thus important mediators used in cosmetics and pharmaceuticals. It is important for liposomes to have small sizes, uniform particle size distribution, and long-term stability. Previously, liposomes have been prepared using a homo mixer, microfluidizer, and horn and bath types of sonicators. However, it is difficult to produce liposomes with small sizes and uniform particle size distribution using these methods. Therefore, we have developed a focused ultrasound method to produce nano-sized liposomes with better size control. In this study, the liposome solutions were prepared using the focused ultrasound method and conventional methods. The liposome solutions were characterized for their size distribution, stability, and morphology. Results showed that the liposome solution prepared using focused ultrasonic equipment had a uniform particle size distribution with an average size of 113.6 nm and a polydispersity index value of 0.124. Furthermore, the solution showed good stability in dynamic light scattering measurements for 4 d and Turbiscan measurements for 1 week.

## 1. Introduction

Phospholipids are bipolar substances consisting of hydrophilic heads and hydrophobic tails [1,2,3]. When a large number of phospholipids are placed in water, liposomes are formed with a structure in which the outside is surrounded by hydrophilic heads and the inside is connected in the form of a phospholipid bilayer sphere with hydrophobic tails [4,5]. This spherical shape has the advantage of capturing all bipolar materials, such as hydrophilic and hydrophobic materials [6]. Owing to these advantages, liposomes can be used in various applications such as cosmetics, medicine, and drug delivery [7,8,9,10].

When liposomes are used as media for cosmetics or drug delivery, the encapsulation efficiency must be optimized to efficiently deliver functional substances. Because encapsulation efficiency is closely related to the size of liposomes, size control is a critical factor in liposome preparation [11]. Liposomes are classified as small unilamellar vesicles (SUV) and large unilamellar vesicles (LUV) based on their size [12,13,14]. If the size of a liposome is too large, the stability of the liposome is reduced, and it cannot properly penetrate the skin epidermis and dermis, thereby reducing the skin absorption ratio. However, if the size is too small, the space to encapsulate the material decreases, reducing the encapsulation efficiency of the functional materials [11,15]. Therefore, it is important to make nano-sized liposomes of an appropriately small size. In addition, the particle size distribution, which indicates the uniformity of the liposome size, is also important. The delivery efficiency of the functional substances increases when the particle size of the liposomes is uniform [16]. Finally, stability is also an important factor that determines whether the liposomes can be preserved for a long period of time [17].

The existing methods for producing liposomes include the method involving the use of a mixer [18] and the high-pressure emulsification method [19,20,21,22]. In addition, sonication using ultrasonic technology is mainly used to produce nano-sized liposomes [23,24,25,26]. Although bath and horn types of sonication apparatuses are commonly used, they present several disadvantages, such as low energy, non-uniform dispersion, and non-adjustable frequency or power of the ultrasonic waves [27]. In this study, we aim to overcome these disadvantages and use a focused ultrasound device that can control frequency and power, thereby producing uniform dispersion.

Focused ultrasound technology effectively utilizes ultrasound energy by focusing it at the center of a cylindrical piezoelectric ceramic [28]. Therefore, the energy distribution is more uniform than that of other ultrasonic equipment, such as bath or horn sonicators. The principle of focused ultrasound technology is based on the effect of cavitation. When ultrasonic waves pass through a liquid medium, microbubbles are formed. The microbubbles grow and shrink repeatedly according to the sound pressure generated by the ultrasonic waves. The bubbles burst when they reach a certain size, releasing a large amount of heat, energy, and pressure [29,30,31]. Through this emitted energy, the particles are physically split to reduce their size [32] or dispersed to prevent aggregation [33,34], thereby increasing stability. In addition, focused ultrasound can control the frequency and power of ultrasonic waves, compensating for the disadvantages of existing ultrasound equipment.

In this study, liposome solutions were prepared using conventional methods such as using a mixer and microfluidizer, sonication methods such as bath and horn types, and focused ultrasound equipment. Subsequently, the size and polydispersity index (PDI) were measured using dynamic light scattering (DLS), the stability was measured using a stability analyzer, and, finally, the shape of the liposomes was observed under a microscope using cryogenic electron microscopy (Cryo-EM) [35,36,37]. Liposomes with smaller and more uniform sizes were expected to be produced through this process.

## 2. Materials and Methods

### 2.1. Materials

A total of 18.2 MΩ distilled (DI) water was used as the aqueous phase, and 95% ethanol (EtOH, Duksan, Ansan-si, Republic of Korea) was used as the oil phase. Among several types of lecithin [38,39], hydrogenated soy lecithin (GL-SPC 75 H, Goshen Biotech, Namyangju-si, Republic of Korea) was chosen to produce phospholipids. Cholesterol (Nippon Fine Chemical, Osaka, Japan) was used as an auxiliary material for strengthening the liposome structure [3,40,41]. Table 1 summarizes the materials used for liposome preparation. 

### 2.2. Methods

#### 2.2.1. Homo Mixer

The aqueous phase, DI water, was heated to approximately 70 °C, and the oil phase, EtOH, in which lecithin and cholesterol were dissolved, was heated to approximately 78 °C. The two solutions were then mixed in a homo mixer (PRIMIX, Awaji, Japan) at 5000 rpm for 20 min.

#### 2.2.2. High-Pressure Emulsification

The aqueous and oil phases were heated in the same way as described in Section 2.2.1. After heating, the aqueous phase was placed into the microfluidics (MF) equipment (Ilsin Autoclave, Daejeon, Republic of Korea), and the oil phase was slowly added using a pipette. After operating the MF equipment to prepare the liposome solution in one pass, the one-pass solution was placed into the MF equipment again. The liposome solution was processed in a total of two passes. 

#### 2.2.3. Bath Sonication

The heating process was the same as that described in Section 2.2.1. After heating, the two solutions were mixed and sonicated for 30 min using a bath sonicator (MUJIGAE, Seoul, Republic of Korea). The ultrasonic frequency was 40 kHz, and the power was approximately 100 W.

#### 2.2.4. Horn Sonication

The heating process was the same as that described in Section 2.2.1. The two solutions were mixed after heating. Because heat was generated when using the horn sonication equipment (Branson Ultrasonics, Brookfield, CT, USA), the mixed solution was immediately placed in an ice bath. The mixed solution was sonicated for 30 min. The ultrasonic frequency was 20 kHz, and the power was approximately 110 W.

#### 2.2.5. Focused Ultrasonication

A high-intensity focused ultrasound device (FS-R01K1, FUST Lab, Daejeon, Republic of Korea) was used for the focused ultrasonic method. The focused ultrasonic equipment consists of a cylindrical piezoelectric ceramic that concentrates ultrasonic waves onto a sample positioned at its center [28]. Accordingly, the sample uniformly absorbs strong mechanical energy, resulting in uniform dispersion forces compared to other ultrasonic equipment. Additionally, the focused ultrasound equipment grants direct control of power and frequency. Hence, in this experiment, liposome production could be optimized by adjusting the power and frequency settings of the focused ultrasonic equipment. Two lead zirconate titanates (PZT) were used to maximize the sonication effect. Both PZT frequencies were 380 kHz [42]; the first PZT had a power of 100 W, and the second PZT had a power of 150 W (Figure S1). The amount and temperature of the aqueous and oil phases were the same as those in the previous experiments. The oil phase was injected at a rate of 1.0 mL/min, and the aqueous phase was injected at a rate of 17.79 mL/min. It took approximately 1 h and 40 min to inject all the oil phase, and the solution was circulated for another 2 h.

### 2.3. Characterization

#### 2.3.1. Visual Confirmation

After liposome solutions were prepared, each solution was placed in a glass bottle. Subsequently, the presence of bubbles and transparency of the liposome solution were observed with the naked eye against a dark background.

#### 2.3.2. Size Distribution

Zetasizer Nano ZSP (Malvern Panalytical, Malvern, UK) is an analytical device capable of measuring the particle size and PDI in a solution. The particle size was analyzed by measuring the scattering intensity over time in a solution under Brownian motion [43,44]. The measurement was performed after diluting the liposome solution 100 times in DI water. The liposome solution was measured daily for 4 d from the day of preparation, and changes in the size and PDI of the liposomes were evaluated.

#### 2.3.3. Stability

Turbiscan AGS (Formulation, Toulouse, France) is a device that can analyze the stability of a solution through changes in aggregation or phase separation by measuring the degree of transmission of the solution at regular time intervals [45]. Stability was analyzed by measuring the liposome solution every 6 h for 1 week.

#### 2.3.4. Cryo-EM

Cryo-EM (Talos L 120C, FEI company, OR, USA) is used to observe frozen biological samples through a transmission electron microscope [46,47]. Unlike other electron microscopes, Cryo-EM has the advantage of easily preventing the samples from deteriorating [48]. The accelerating voltage was 120 kV, and the ice growth rate was less than 0.7 nm/h.

## 3. Results

### 3.1. Size Distribution

**Figure 1:**
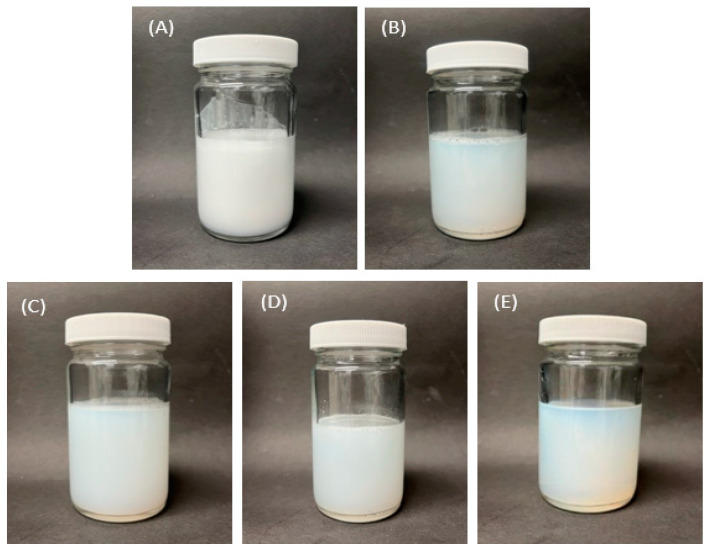
Liposome solutions prepared using (**A**) homo mixer, (**B**) microfluidizer, (**C**) bath sonication, (**D**) horn sonication, and (**E**) focused ultrasonication.

Figure 1 shows photographs of the prepared liposome solutions. The liposome solution prepared using the homo mixer was opaque with numerous bubbles. In contrast, the liposome solutions prepared using MF, bath sonication, horn sonication, and focused ultrasonication had no bubbles and were more transparent. In the case of the liposome solution prepared using focused ultrasonication, there were no bubbles in the upper layer of the solution, and the color of the solution was the most transparent. Transparency is a correlation between particle size and light scattering, with larger particles scattering more light and smaller particles scattering less light [49].

**Figure 2:**
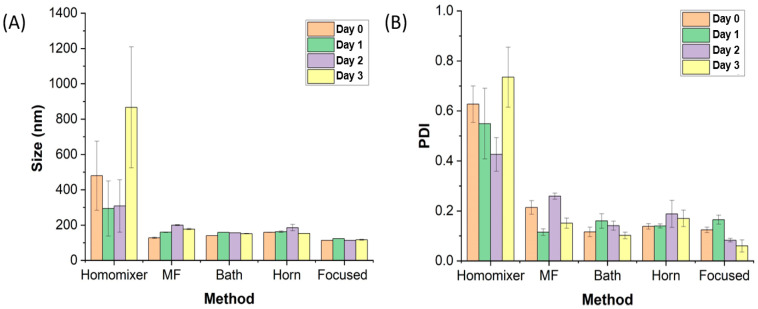
Changes in (**A**) size and (**B**) PDI of the liposomes prepared using different methods, measured for 4 d.

Figure 2 shows the size and size distribution results measured using DLS, and Table 2 lists the size and PDI. As expected, the liposome solution prepared using the homo mixer had a much larger size and PDI value than those of other liposome solutions. In addition, in terms of the stability measurement taken 3 d after the experiment, the size change was large, making it highly unstable. The liposome solution prepared using the MF method consisted of relatively small liposomes (127.8 nm on the day of the experiment); however, it was difficult to treat it as a stable solution because the size increased to 199.6 nm after 2 d. The solution prepared using bath sonication was judged to be relatively stable, considering that the liposome size was measured to be near 150 nm for 3 d. The size of the liposomes prepared using horn sonication increased from 159.2 nm to 185.8 nm on Day 2 and then decreased to 153.1 nm, showing an unstable appearance. In addition, its PDI value was 0.138 on the day of the experiment, and it increased over time. Finally, in the case of the liposome solution prepared using the focused ultrasound method, the liposome size measured on the day of the experiment was the smallest (113.6 nm), and the solution also seemed to be considerably stable as a result of the measurement taken after a total of 4 d. In terms of the PDI, the size distribution seemed to be highly uniform, as the PDI value was measured to be approximately 0.1.

### 3.2. Stability

**Figure 3:**
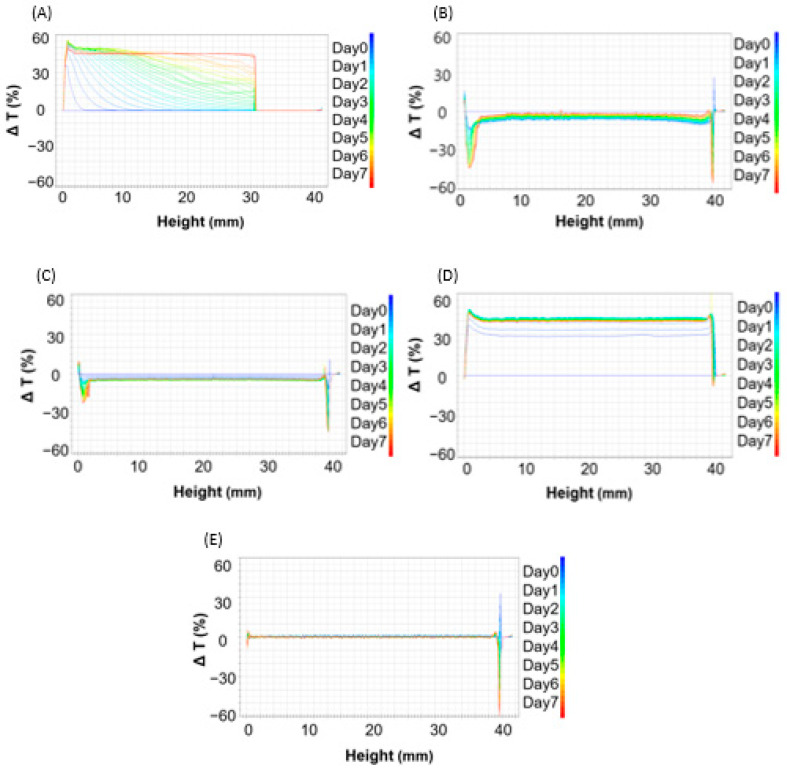
Delta transmission of liposome solutions prepared using (**A**) homo mixer, (**B**) microfluidizer, (**C**) bath sonication, (**D**) horn sonication, and (**E**) focused ultrasonication.

Figure 3 shows the result of the Turbiscan measurement of the degree of delta transmission every 6 h for 1 week, which is an important result indicating the stability of the liposome solution. The x-axis represents the height of the bottle containing the sample, and the y-axis represents the delta transmission change (%). First, in the case of the solution prepared using a homo mixer, the delta transmission percentage was approximately 50% or higher, indicating that many liposome particles aggregated in the solution. Next, the liposome solution prepared using MF was less unstable than that prepared using the homo mixer, but it seems that some aggregation and phase separation occurred. In the case of the solution prepared using bath sonication, a graph in the form of phase separation was observed at the bottom and top of the solution, and the particles aggregated. However, its delta transmission percentage was approximately 10%, indicating that it was more stable than the MF liposome solution. Next, in the case of the liposome solution prepared using horn-type sonication, it appears that the delta transmission percentage was high across the whole solution, indicating aggregation. Finally, the solution prepared using the focused ultrasound device had the lowest degree of data transmission, indicating that it was considerably stable and did not show much aggregation or phase separation compared to other liposome solutions.

### 3.3. Cryo-EM

**Figure 4:**
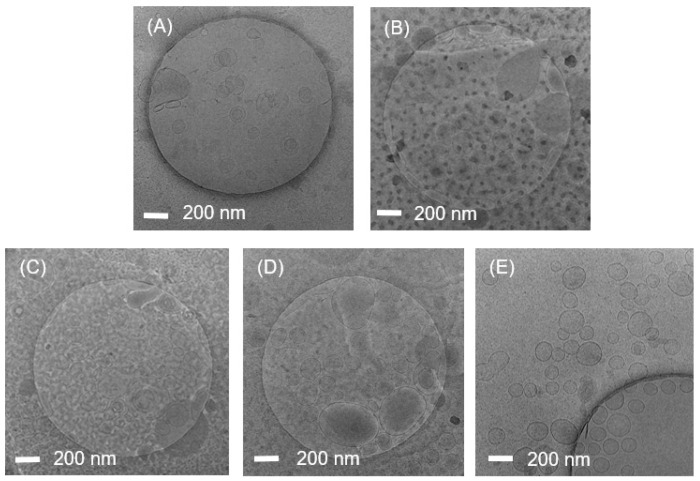
Cryo-EM images of liposome solutions prepared using (**A**) homo mixer, (**B**) microfluidizer, (**C**) bath sonication, (**D**) horn sonication, (**E**) focused ultrasonication.

Figure 4 shows Cryo-EM images of the liposomes. The circular shape shown in the images is a grid appearing on the Cryo-EM measuring plate. The concentration of liposomes in the solution prepared using the homo mixer was remarkably low. This is because the liposome solution was phase-separated, and many bubbles were generated. In the case of the liposome solution prepared using the MF equipment, the size of liposomes was non-uniform, although most liposomes had a large size of approximately 200 nm. The liposome solution prepared using bath sonication also exhibited non-uniform particle sizes, and a significant number of double-layer or multi-layer liposomes were observed. Next, the liposome solution prepared using horn sonication produced many large liposomes with non-uniform particle sizes. Finally, the liposome solution prepared using focused ultrasound showed liposomes with a size smaller than 200 nm; however, the size distribution was not very uniform. This result demonstrated that the focused ultrasound method was capable of producing smaller liposomes than those produced using other methods. 

**Figure 5:**
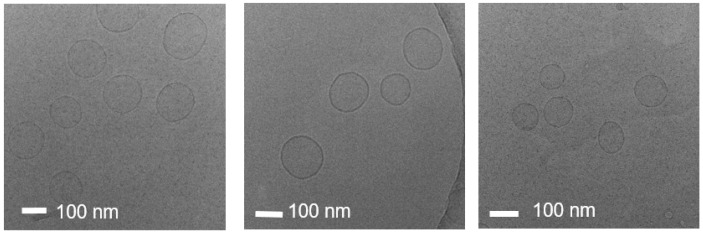
Cryo-EM images of liposomes prepared using focused ultrasound method.

Figure 5 shows the Cryo-EM images of the liposome solution prepared using the focused ultrasound method. Compared to the liposomes prepared using other methods, which had a size of approximately 200 nm, the liposomes prepared using focused ultrasound had a size of approximately 100 nm. In addition, it was confirmed that single-layer liposomes with uniform shape and size distribution were produced. 

## 4. Discussion

In this study, liposomes were prepared using five different methods. First, the mixer manufacturing approach, being a simple mixing method, generated numerous bubbles, leading to larger size and PDI values in DLS measurements. Next, the high-pressure emulsification method, widely employed in liposome preparation, was found to be relatively simple and time-consuming but lacked stability. Among the ultrasonic methods used to reduce the size of liposomes, bath sonication showed minimal change in size during the 4-day measurement period. However, the size itself did not reach the desired small size, which was considered a disappointing result. Similarly, horn sonication yielded a size similar to bath sonication, albeit slightly less stable. This could be attributed to the uneven concentration of ultrasonic energy within the liposome solution. Finally, the focused ultrasonic method proved to be the most promising. It not only resulted in the smallest size and uniform particle distribution, but also demonstrated superior stability compared to other methods. 

As noted in the Introduction, liposomes become more stable as their size decreases. Achieving a uniform and small size enhances the efficiency of collecting functional materials. Upon analyzing the above results, it is evident that the focused ultrasonication method yielded liposomes with the smallest and most uniform size, compared to simple techniques like homogenization and high-pressure emulsification, as well as existing sonication methods such as bath-type and horn-type methods.

This favorable outcome is attributed to the focused ultrasound method’s ability to apply appropriate frequency and energy to the liposome solution while also directing concentrated energy to the circulating solution. 

Consequently, employing the focused ultrasound method is expected to lead to the creation of nano-sized liposomes with uniform structures, enhanced stability, and significantly improved encapsulation efficiency. These liposomes are likely to find applications in diverse fields such as drug delivery and cosmetics.

## 5. Conclusions

In this study, liposomes with a small particle size and uniform size distribution were prepared using focused ultrasound technology and compared with the liposomes prepared using the existing manufacturing methods, such as homo mixer, microfluidizer, and bath and horn types of sonication. The liposomes prepared using focused ultrasound equipment had an average size of 113.6 nm and a PDI of 0.124, demonstrating a relatively small particle size and uniform size distribution. In addition, the stability measurement up to 3 d after the experiment showed little size change, and the size was 123.8 nm, 113.7 nm, and 116.5 nm, on Days 1, 2, and 3, respectively. This level of stability surpassed that of solutions prepared using other methods. Therefore, it was confirmed that this focused ultrasound method was more effective than the existing liposome preparation methods. This focused ultrasound technology can be applied to produce stable liposome solution with small and uniform sizes for applications in various fields, such as cosmetics, food, medicine, and drug delivery.

## Figures and Tables

**Table 1:** Properties of materials for liposome solution preparation.

Material	EtOH	Lecithin	Cholesterol	DI Water
Property	95%	Phosphatidylcholine ≥ 75%	Molecular weight: 386.65	Resistance: 18.2 MΩ
Amount	Volume: 70 mL	Weight: 1.0 g	Weight: 0.3 g	Volume: 100 mL

**Table 2:** Particle size and size distribution for 4 d.

		Day 0	Day 1	Day 2	Day 3
Homo mixer	Size (nm) (S.D value)	479.2 (195.9)	293.9 (156.2)	308.3 (148.4)	866.8 (342.5)
	PDI (S.D value)	0.627 (0.073)	0.549 (0.141)	0.426 (0.067)	0.735 (0.12)
Microfluidizer	Size (nm) (S.D value)	127.8 (3.523)	160 (1.305)	199.6 (2.307)	177.6 (3.536)
	PDI (S.D value)	0.214 (0.027)	0.115 (0.013	0.259 (0.012)	0.151 (0.02)
Bath sonication	Size (nm) (S.D value)	140.1 (0.8544)	158.4 (0.7767)	155.9 (0.8327)	151.3 (1.464)
	PDI (S.D value)	0.116 (0.019)	0.160 (0.029)	0.141 (0.018)	0.102 (0.013)
Horn sonication	Size (nm) (S.D value)	159.2 (1.212)	162.5 (4.605)	185.8 (17.66)	153.1 (1.058)
	PDI (S.D value)	0.138 (0.011)	0.140 (0.008)	0.188 (0.054)	0.170 (0.033)
Focused ultrasound	Size (nm) (S.D value)	113.6 (0.3055)	123.8 (0.3055)	113.7 (0.8327)	116.5 (2.371)
	PDI (S.D value)	0.124 (0.011)	0.165 (0.018)	0.083 (0.007)	0.060 (0.024)

## Data Availability

Not applicable.

## Supplementary Materials

The following supporting information can be downloaded at: https://www.mdpi.com/article/10.3390/nano13192618/s1, Figure S1: Changes in size over 4 d with PZT power (W).
