# Supplementary material for: Preparation of Uniform Nano Liposomes Using Focused Ultrasonic Technology
Source: Nanomaterials (Basel). 2023 Sep 22;13(19):2618. doi: 10.3390/nano13192618 (PMC10574396; doi:10.3390/nano13192618)
Supplement: Supplementary file 1 [file nanomaterials-13-02618-s001.zip › nanomaterials-2621291-supplementary.pdf]

nanomaterials

Article

Preparation of Uniform Nano Liposomes Using Focused Ultrasonic Technology

Ji-Soo Yun [1,2], Seon-Ae Hwangbo [1,*] and Young-Gyu Jeong [2,*]

[1] Nanosafety Team, Safety Measurement Institute, Korea Research Institute of Standards and Science (KRISS), 267 Gajeong-ro, Yuseong-gu, Daejeon 34113, Republic of Korea; jisoo991208@kriss.re.kr

[2] Department of Applied Organic Materials Engineering, Chungnam National University, Daejeon 34134, Republic of Korea

* Correspondence: hbsa.kriss@gmail.com (S.-A.H.); ygjeong@cnu.ac.kr (Y.-G.J.)

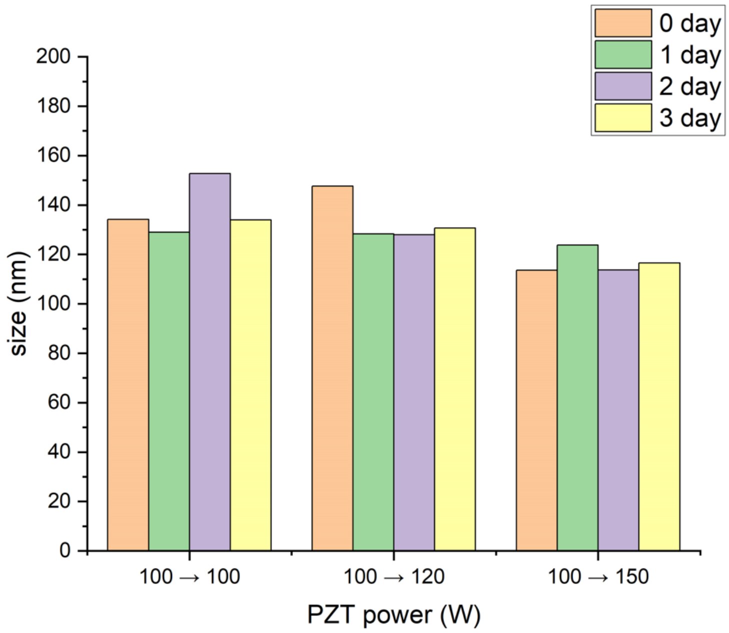

Figure S1. Changes in size over 4 d with PZT power (W).
